# Histone Deacetylase 3 Regulates Adipocyte Phenotype at Early Stages of Differentiation

**DOI:** 10.3390/ijms22179300

**Published:** 2021-08-27

**Authors:** Dalma Cricrí, Lara Coppi, Silvia Pedretti, Nico Mitro, Donatella Caruso, Emma De Fabiani, Maurizio Crestani

**Affiliations:** Dipartimento di Scienze Farmacologiche e Biomolecolari, Università degli Studi di Milano, Via Balzaretti 9, 20133 Milano, Italy; dalma.cricri@unimi.it (D.C.); lara.coppi@unimi.it (L.C.); silvia.pedretti@unimi.it (S.P.); nico.mitro@unimi.it (N.M.); donatella.caruso@unimi.it (D.C.)

**Keywords:** epigenetics, obesity, HDAC3, adipocytes, differentiation, metabolism, inflammation

## Abstract

Obesity is a condition characterized by uncontrolled expansion of adipose tissue mass resulting in pathological weight gain. Histone deacetylases (HDACs) have emerged as crucial players in epigenetic regulation of adipocyte metabolism. Previously, we demonstrated that selective inhibition of class I HDACs improves white adipocyte functionality and promotes the browning phenotype of murine mesenchymal stem cells (MSCs) C3H/10T1/2 differentiated to adipocytes. These effects were also observed in *db/db* and diet induced obesity mouse models and in mice with adipose-selective inactivation of HDAC3, a member of class I HDACs. The molecular basis of class I HDACs action in adipose tissue is not deeply characterized and it is not known whether the effects of their inhibition are exerted on adipocyte precursors or mature adipocytes. Therefore, the aim of the present work was to explore the molecular mechanism of class I HDAC action in adipocytes by evaluating the effects of HDAC3-specific silencing at different stages of differentiation. HDAC3 was silenced in C3H/10T1/2 MSCs at different stages of differentiation to adipocytes. shRNA targeting HDAC3 was used to generate the knock-down model. Proper HDAC3 silencing was assessed by measuring both mRNA and protein levels of mouse HDAC3 via qPCR and western blot, respectively. Mitochondrial DNA content and gene expression were quantified via qPCR. HDAC3 silencing at the beginning of differentiation enhanced adipocyte functionality by amplifying the expression of genes regulating differentiation, oxidative metabolism, browning and mitochondrial activity, starting from 72 h after induction of differentiation and silencing. Insulin signaling was enhanced as demonstrated by increased AKT phosphorylation following HDAC3 silencing. Mitochondrial content/density did not change, while the increased expression of the transcriptional co-activator *Ppargc1b* suggests the observed phenotype was related to enhanced mitochondrial activity, which was confirmed by increased maximal respiration and proton leak linked to reduced coupling efficiency. Moreover, the expression of pro-inflammatory markers increased with HDAC3 early silencing. To the contrary, no differences in terms of gene expression were found when HDAC3 silencing occurred in terminally differentiated adipocyte. Our data demonstrated that early epigenetic events mediated by class I HDAC inhibition/silencing are crucial to commit adipocyte precursors towards the above-mentioned metabolic phenotype. Moreover, our data suggest that these effects are exerted on adipocyte precursors.

## 1. Introduction

Overweight and obesity are defined by the World Health Organization as abnormal or excessive fat accumulation that presents a risk to health. Patients are classified as overweight when the body mass index (BMI) is above 25 kg/m^2^ or obese if above 30 kg/m^2^ [1]. The prevalence of these conditions is climbing worldwide; in 2016, 39% of adults were overweight and 13% were obese [2]. They represent primary risk factors for a broad range of severe pathologies such as cardiovascular disease [3], type 2 diabetes [4], musculoskeletal disorders [5] and cancer [6]. Despite the increasing prevalence, available strategies for prevention and treatment of obesity have shown mild effectiveness to date [7]. Thus, further investigations on pathogenesis mechanisms and potential therapeutic approaches are required for lowering the risks of an overweight and obesity pandemic and the related comorbidities.

In addition, overweight and obesity are associated with low-grade chronic inflammation mainly related to immune dysregulation within adipose tissue [8]. M1 pro-inflammatory macrophages are the most abundant immune cells accumulating and releasing pro-inflammatory cytokines that contribute to the development of obesity complications [9]. On the other hand, pro-inflammatory stimuli have also been shown to be essential for adipogenesis and adipose tissue physiology. Adipocyte metabolism and functionality are affected in a mouse model with adipose tissue-specific reduction of inflammation. As a consequence of reduced adipogenesis, fat storage capacity decreases and results in ectopic fat accumulation, hepatic steatosis and glucose intolerance [10].

Adipose tissue mass is consequent to cell proliferation and differentiation of precursors to mature adipocytes. This represents a physiological process essential to adipose tissue development and turnover [11]. Moreover, a pool of multipotent stromal vascular cells populates adipose tissue guaranteeing tissue renewal [12], while the regulation of this process is altered in overweight or obese subjects [13].

Differentiation of precursor cells to adipocytes is highly regulated by a transcriptional cascade that directs gene expression programming [14]. This process is defined as adipogenesis and it is activated by a complex cascade of transcriptional factors. The first wave of activation is initiated by CREB (cAMP response element binding protein) and members of the C/EBP (CCAAT/enhancer-binding protein) family such as C/EBPβ. Moreover, other early adipogenic factors such as SREBP-1c (sterol regulatory element binding protein-1c) play a relevant role during this stage [15]. Their action determines a second wave of transcriptional activation, mainly mediated by the nuclear receptor peroxisome proliferator-activated receptor γ (PPARγ). Furthermore, C/EBPα is an important late adipogenic factor stimulating and amplifying PPARγ action [16].

Adipose tissue is classified in white adipose tissue (WAT) and brown adipose tissue (BAT) [17]. BAT originates during embryonal development and it is mainly involved in heat production due to uncoupling protein 1 (UCP1) action, which uncouples the respiratory chain within the inner mitochondria membrane from ATP production [18]. In contrast, WAT is primarily important for energy storage under the form of triacylglycerols [19]. Furthermore, WAT covers relevant endocrine functions such as glucose homeostasis regulation through adipokines release [20]. BAT and WAT also have significant morphologic differences. In fact, brown adipocytes are characterized by a high number of mitochondria with intense oxidative activity and interspersed among multilocular lipid droplets; white adipocytes typically present unilocular droplets where triglycerides are stored [21]. Beside white and brown adipocytes, beige adipocytes present intermediate characteristics. The process related to cell differentiation to brown-like phenotype is defined as “browning” [22].

Epigenetic modifications modulate gene expression by acting on chromatin remodeling [23]. Epigenome modifiers have been shown to play a crucial role in adipose tissue metabolism, predisposition and development of obesity [24,25,26]. Histone deacetylases (HDACs) catalyze the removal of acetyl groups from histones promoting chromatin condensation and reducing gene expression. To date, four different classes of HDACs have been described. Class I histone deacetylases includes HDAC 1, 2, 3 and 8, all localized in the nucleus and with pronounced activity on histones; their expression is ubiquitarian. Class II HDACs (HDAC 4, 5, 6, 7, 9, 10) shuttle between nucleus and cytoplasm and do not have marked activity on histones. Class III is represented by sirtuins, and class IV includes only HDAC11, which does not have a clear role [27].

By using both genetic and diet-induced obese mouse models, we previously showed that the selective inhibition of class I HDACs with the small molecule MS-275 improved obese phenotype and insulin sensitivity in vivo [28,29]. Moreover, in vitro MS-275 stimulates adipocyte metabolism promoting expression of genes related to adipocyte differentiation and functionality, including *Pparg*, a master regulator of the adipocyte phenotype [30], browning (*Ucp1*) and oxidative metabolism that supports thermogenesis. Knockout in adipose tissues in mice and shRNA-mediated silencing in adipocyte cultures of HDAC3 recapitulated browning and the metabolic rewiring observed with the HDAC inhibitor MS-275 [31]. However, it should be noted that the effects of HDAC inhibitors may not necessarily overlap those deriving from genetic ablation or silencing of a specific HDAC. HDAC inhibitors reduce the catalytic activity of these proteins, while they may not affect other possible features of the same proteins that are not linked to their catalytic activity. Thus, genetic ablation or silencing of a specific HDAC could lead to results that do not completely overlap with those obtained with a chemical inhibitor. As an example, HDAC3 has been shown to act as a coactivator, independently from its catalytic activity, in macrophages, where it activates the expression of inflammatory genes via a non-canonical mechanism [32]. Therefore, it is crucial to assess the role of HDAC3 in the adipocyte phenotype by testing the effects of its silencing, as opposed to chemical inhibition. Moreover, to further explore whether a specific class I HDAC regulates adipose tissue functions at different stages of differentiation, we asked whether depletion of HDAC3 affects adipocyte precursors or mature adipocytes. Our results indicate that the main effects were achieved only when HDAC3 expression was reduced at an early stage of adipocyte differentiation, while depletion of HDAC3 in mature adipocytes did not lead to major changes of their phenotype.

## 2. Results

### 2.1. Early Silencing of HDAC3 Amplifies Adipocyte Differentiation and Functionality

**Figure 1:**
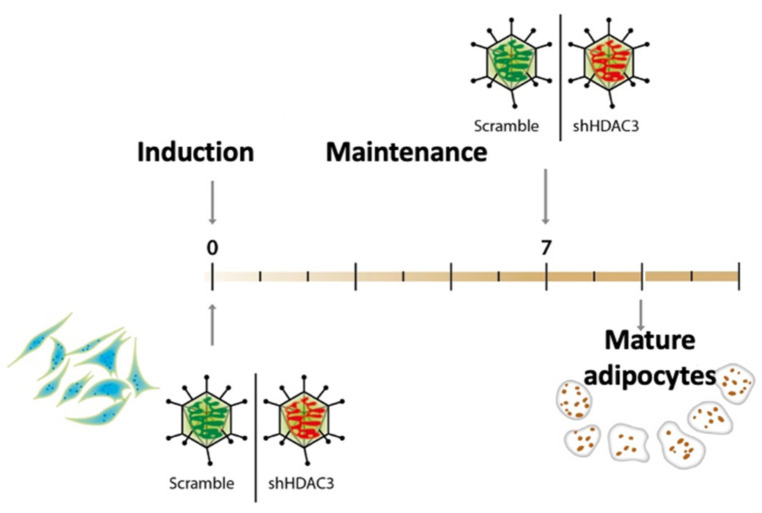
C3H/10T1/2 cells induced to differentiate to adipocytes using a standard hormonal cocktail and maintained until terminally differentiated. HDAC3 was silenced by adenovirus-mediated short hairpin RNA interference at the beginning of differentiation (day 0) or at the end of differentiation (day 7).

**Figure 2:**
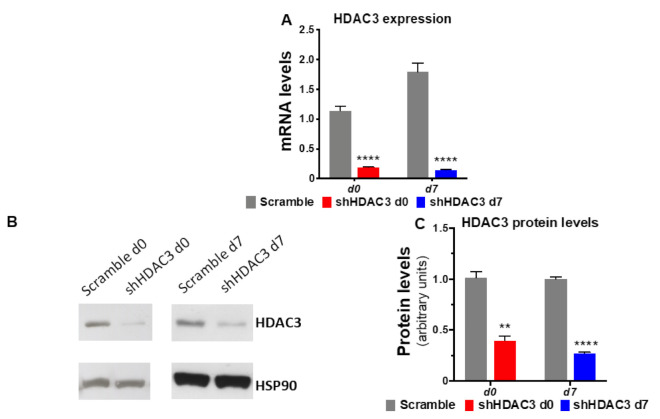
Silencing of HDAC3 confirmed at mRNA and at protein level. (**A**) mRNA expression data are presented as mean ± SEM (*n* = 9 from 3 independent experiments). (**B**) Representative western blot of HDAC3 protein levels from three independent experiments. (**C**) Quantitation of HDAC3 protein levels in C3H/10T1/2 adipocytes silenced at day 0 or at day 7 of differentiation (*n* ≥ 3 from 3 independent experiments). Cells silenced at day 0 were harvested at day 9 after induction of differentiation, while cells silenced at day 7 were harvested at day 11 after induction of differentiation. Statistical analyses were performed using unpaired Student’s *t*-test, ** *p* < 0.01 and **** *p* < 0.0001, respectively.

To gain insights into the molecular basis of class I HDACs in adipose tissue and establish whether the phenotypic effects observed using the class I HDAC inhibitor MS-275 were related to precursors or mature adipocytes, MSCs were induced to differentiate to adipocytes and HDAC3 was silenced at the beginning (day 0) and at the end (day 7) of the differentiation, as shown in the scheme in Figure 1. Silencing was confirmed both at mRNA and protein level (Figure 2).

**Figure 3:**
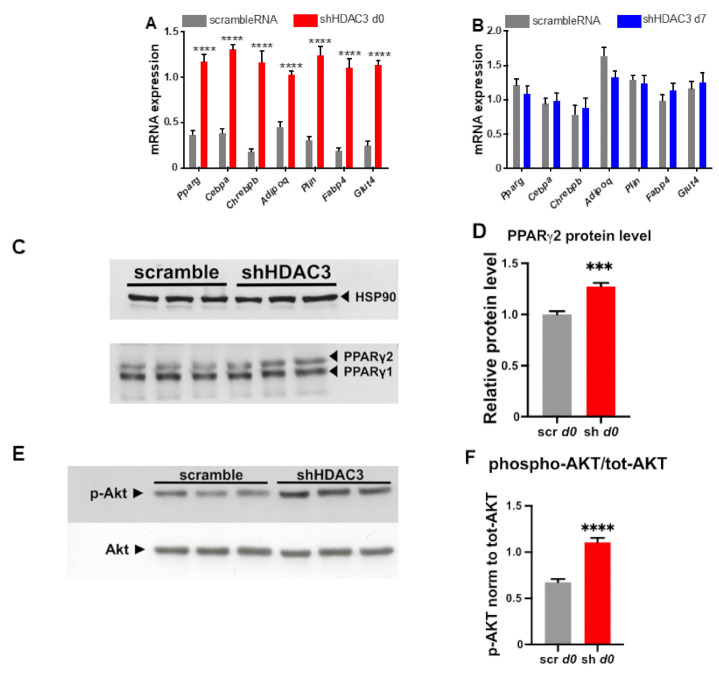
Expression of genes regulating adipocyte differentiation and functionality in C3H/10T1/2 cells infected with scramble/shHDAC3 at day 0 (**A**) or at day 7 (**B**) of differentiation to adipocytes. Cells silenced at day 0 were harvested at day 9 after induction of differentiation, while cells silenced at day 7 were harvested at day 11 after induction of differentiation. Data are presented as mean ± SEM. Statistical analyses were performed with GraphPad PRISM using unpaired Student’s *t*-test, **** *p* < 0.0001 (*n* ≥ 8 from 3 independent experiments). (**C**) Representative western blot of PPARγ from C3H/10T1/2 cells infected with scramble/shHDAC3 at d0 of differentiation to adipocytes and (**D**) quantification of the protein bands in panel (**C**); data are presented as mean ± SEM; statistical analysis was performed with GraphPad PRISM using unpaired Student’s *t*-test, *** *p* < 0.001 (*n* = 6 from 2 independent experiments). (**E**) Representative western blot of C3H/10T1/2 cells infected with scramble/shHDAC3 at d0 of differentiation to adipocytes for detection of phosphorylated Akt (p-Akt) vs. total Akt (Akt); HSP90 was used as a loading control (not shown). (**F**) Quantification of p-Akt/tot-Akt ratio from western blot in panel (**E**); data are presented as mean ± SEM; statistical analysis was performed with GraphPad PRISM using unpaired Student’s *t*-test, **** *p* < 0.0001 (*n* = 6 from 2 independent experiments).

HDAC3 silencing at day 0 significantly increased the expression of genes important for adipocyte differentiation and functionality. In particular, the mRNA levels of *Pparg*, *Cebpa* and carbohydrate-responsive element-binding protein β (*Chrebpb*), key transcriptional regulators of adipogenesis, increased following HDAC3 silencing at d0 of differentiation (Figure 3A), while no change at the mRNA levels of these transcription factors was noted when HDAC3 was silenced at d7 of differentiation (Figure 3B). Accordingly, the protein level of PPARγ2, the receptor isoform specific to adipocytes, was higher, whereas the PPARγ1 protein level did not change (Figure 3C,D). Since *Chrebpb* expression has been shown to predict insulin sensitivity through upregulation of de novo lipogenesis in adipose tissue [33], we were prompted to test the insulin receptor cascade activation in C3H/10T1/2 adipocytes in which we silenced HDAC3. As shown in Figure 3E,F, western blot analysis indicates that early silencing of HDAC3 increases AKT phosphorylation, a known marker of insulin receptor activation.

**Figure 4:**
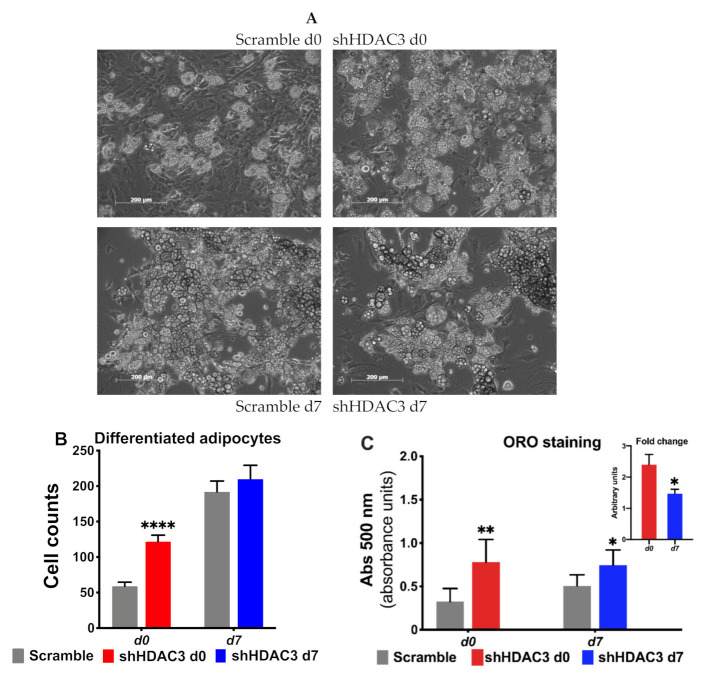
Early silencing of HDAC3 increases the number of differentiated adipocytes. (**A**) Representative images of C3H/10T1/2 MSCs with HDAC3 silencing vs. scramble controls at day 0 or day 7 of differentiation. (**B**) Quantification of mature adipocytes with HDAC3 silencing at day 0 or day 7 post induction of differentiation. Cells silenced at day 0 were harvested at day 9 after induction of differentiation, while cells silenced at day 7 were harvested at day 11 after induction of differentiation. Statistical analysis was performed with GraphPad PRISM using unpaired Student’s *t*-test, **** *p* < 0.0001 (*n* ≥ 8 counted fields/condition). (**C**) Oil Red O (ORO) staining to quantify lipid content in response to HDAC3 silencing. Inset shows the different fold change of lipid content induced when HDAC3 was silenced at d0 vs. d7 post induction of differentiation. Statistical analysis was performed with GraphPad PRISM using unpaired Student’s *t*-test, * *p* < 0.05 and ** *p* < 0.01 (*n* = 6).

We also detected increased expression of several markers of adipocyte functionality with HDAC3 early silencing. For instance, the expression of lipid droplet-associated protein perilipin (*Plin*), fatty acid-binding protein 4 (*Fabp4*), adiponectin (*Adipoq*) and glucose transporter 4 (*Glut4*) increased (Figure 3A), suggesting amplified adipogenic potential (Figure 3A). In contrast, when HDAC3 was silenced in mature adipocytes, the expression profile of the same genes was not affected (Figure 3B). The results of the expression of genes relevant for adipocyte differentiation were consistent with a greater number of differentiated adipocytes when HDAC3 was silenced at day 0 vs. cells treated with scramble RNA (Figure 4A), while no difference was detected in the number of differentiated adipocytes when HDAC3 was silenced at day 7 vs. cells treated with scramble RNA (Figure 4B). Oil Red O (ORO) staining confirmed that HDAC3 silencing at d0 of differentiation increased lipid content in adipocytes, whereas the increase of lipid content was significantly lower with HDAC3 silencing at d7 (2.5-fold with HDAC3 silencing at d0 vs. 1.5-fold change with HDAC3 silencing at d7, Figure 4C and inset).

### 2.2. Early Silencing of HDAC3 Promotes Oxidative Metabolism of Mature Adipocytes

**Figure 5:**
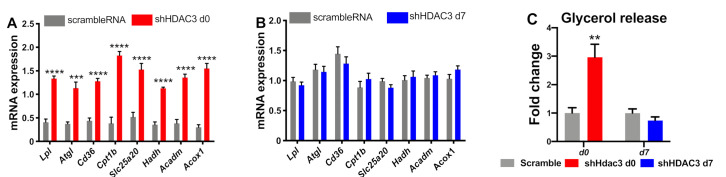
Expression of genes relevant for lipid mobilization and fatty acid β-oxidation in C3H/10T1/2 cells infected with scramble/shHDAC3 at day 0 (**A**) or at day 7 (**B**) of differentiation to adipocytes. Glycerol release from adipocytes in the culture medium (**C**). Cells silenced at day 0 were harvested at day 9 after induction of differentiation, while cells silenced at day 7 were harvested at day 11 after induction of differentiation. Data are presented as mean ± SEM. Statistical analysis was performed with GraphPad PRISM using unpaired Student’s *t*-test, ** *p* < 0.01, *** *p* < 0.001, **** *p* < 0.0001 (*n* ≥ 6 from at least 2 independent experiments).

To further characterize the effect of HDAC3 silencing at day 0 or day 7 of differentiation on the phenotype of C3H/10T1/2 adipocytes, we extended the analysis to genes involved in oxidative metabolism. We focused on the expression of genes regulating fatty acid uptake, lipolysis and β-oxidation. HDAC3 silencing at day 0 increased the expression of the cluster of differentiation 36 (*Cd36*), lipoprotein lipase (*Lpl*) and adipose triglyceride lipase (*Atgl*), carnitine palmitoyl-transferase 1b (*Cpt1b*), carnitine-acylcarnitine translocase *Slc25a20* (solute carrier family 25 member 20), hydroxyacyl-coenzyme A dehydrogenase (*Hadh*), medium-chain acyl-CoA dehydrogenase (*Acadm*) and peroxisomal acyl-coenzyme A oxidase 1 (*Acox1*) (Figure 5A). These results suggest enhanced lipid mobilization and mitochondrial fatty acid β-oxidation. These are crucial metabolic pathways that support browning of adipocytes. In contrast, HDAC3 silencing at day 7 did not affect these pathways as the expression profile of these genes was not notably altered (Figure 5B). Increased lipid catabolic activity in adipocytes with HDAC3 silencing at d0 was confirmed by measuring glycerol release in the culture medium, while no difference was observed with HDAC3 silencing at d7 (Figure 5C).

### 2.3. Early Silencing of HDAC3 Stimulates Adipocyte Browning and Mithocondria Functionality

**Figure 6:**
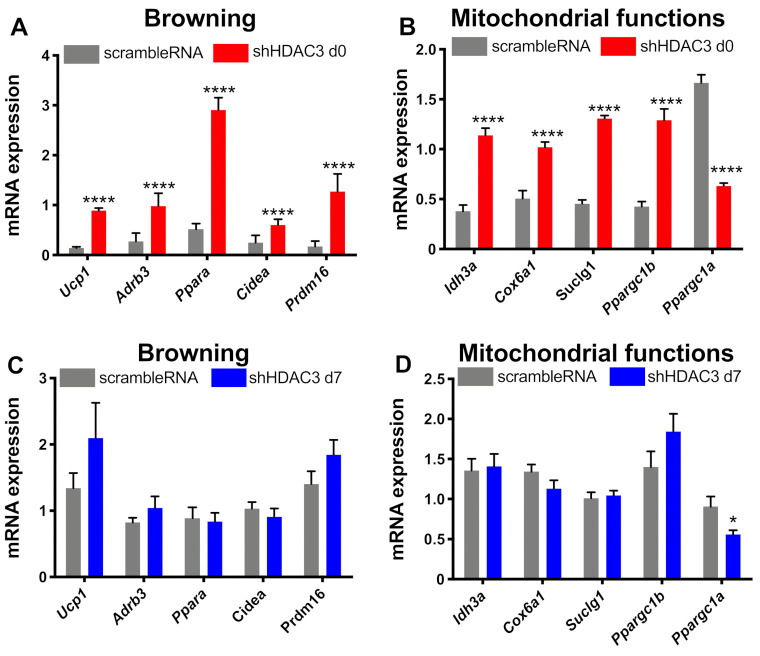
Expression of genes related to browning and mitochondrial functions. C3H/10T1/2 cells were infected with scramble/shHDAC3 at day 0 and expression of genes for browning (**A**) and mitochondrial functions (**B**) of differentiation to adipocytes. The expression of the same genes was measured in C3H/10T1/2 cells infected with scramble/shHDAC at day 7 (**C**,**D**). Cells silenced at day 0 were harvested at day 9 after induction of differentiation, while cells silenced at day 7 were harvested at day 11 after induction of differentiation. Data are presented as mean ± SEM. Statistical analyses were performed with GraphPad PRISM and unpaired Student’s *t*-test, **** *p* < 0.0001 (*n* ≥ 8 from 3 independent experiments).

The expression of genes involved in browning and mitochondrial functionality was also analyzed. HDAC3 silencing at day 0 significantly increased the expression of genes involved in browning, in particular uncoupling protein 1 (*Ucp1*), β_3_ adrenergic receptor (*Adrb3*), peroxisome proliferator activator receptor α (*Ppara*), cell death inducing DFFA-like effector α (*Cidea*), typically expressed in brown adipose tissue, and PR domain containing 16 (Prdm16), a transcriptional co-regulator of brown adipocytes differentiation (Figure 6A). However, the expression of these genes did not change when HDAC3 silencing was elicited at day 7 (Figure 6C). Moreover, HDAC3 silencing at the beginning of differentiation showed higher expression of genes important for mitochondrial functions and metabolism. In fact, the expression of isocitrate dehydrogenase subunit α (*Idh3a*), cytochrome c oxidase subunit 6a1 (*Cox6a1*), succinyl-CoA ligase subunit 1 (*Suclg1*) and peroxisome PPARγ coactivator 1β (*Ppargc1b*) was significantly amplified (Figure 6B). On the contrary, the mRNA levels of peroxisome PPARγ coactivator 1α (*Ppargc1a*), master regulator of mitochondria biogenesis, significantly decreased (Figure 6B). Still, the differences of gene expression were absent when HDAC3 silencing was performed in differentiated adipocytes (Figure 6C,D), apart from *Ppargc1a*, whose expression was reduced, although to a lower extent than in cells with HDAC3 silencing at day 0 (Figure 6D).

**Figure 7:**
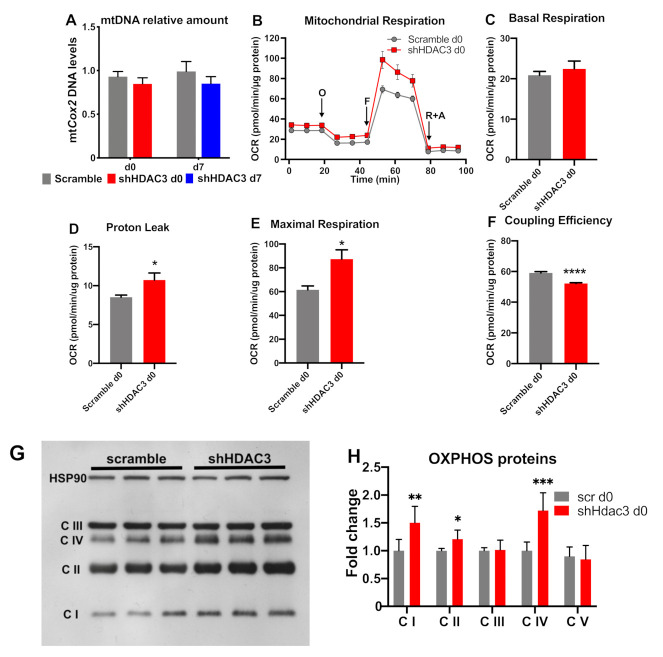
mtDNA levels and mitochondrial respiration in C3H/10T1/2 adipocytes infected with scramble/shHDAC3. (**A**) Cells silenced at day 0 were harvested at day 9 after induction of differentiation, while cells silenced at day 7 were harvested at day 11 after induction of differentiation (*n* = 18 from 6 independent experiments). Cells silenced at day 0 were analyzed at day 9 after induction of differentiation for mitochondrial respiration (O, oligomycin addition; F, FCCP addition; rotenone + antimycin A addition) (**B**); basal respiration (**C**), proton leak (**D**), maximal respiration (**E**) and coupling efficiency (**F**) were calculated. Data are presented as mean ± SEM. Statistical analyses were performed with GraphPad PRISM and unpaired Student’s *t*-test, * *p* < 0.05 and **** *p* < 0.0001 (*n* = 5). (**G**) Representative western blot of C3H/10T1/2 cells infected with scramble/shHDAC3 at d0 of differentiation to adipocytes to detect OXPHOS proteins: C I, complex I; C II, complex II; C III, complex III; C IV, complex IV; complex V (ATP synthase) was visible only at longer exposure of the film; however, the level of this complex did not differ between scramble and shHDAC3 (not shown). (**H**) Quantification of OXPHOS proteins from western blot in panel (**G**); data are presented as mean ± SEM; statistical analysis was performed with GraphPad PRISM using unpaired Student’s *t*-test, * *p* < 0.05, ** *p* < 0.01 and *** *p* < 0.001 (*n* = 6 from 2 independent experiments).

Accordingly, mitochondrial DNA of cytochrome c oxidase subunit 2 (mtCox2) did not increase with HDAC3 silencing at day 0 (Figure 7A). When we measured mitochondrial functionality (Figure 7B), despite no change in basal respiration (Figure 7C), we found an increase in proton leak (Figure 7D) and maximal respiration (Figure 7E), linked to reduced coupling efficiency (Figure 7F) in HDAC3-depleted adipocytes. Furthermore, the higher expression of OXPHOS proteins (i.e., complex I, complex II and complex IV) was consistent with the increased oxidative metabolism of silenced adipocytes (Figure 7G,H).

Overall, these results indicate that silencing of HDAC3 leads to browning of mature adipocytes, with enhanced uncoupled respiration and greater capacity of oxidative metabolism. Notably, these observations also suggest HDAC3 silencing has an impact on adipocyte differentiation and metabolism regulating the early stages of adipogenesis.

### 2.4. Early Silencing of HDAC3 Modulates Adipocyte Pro-Inflammatory Profile

**Figure 8:**
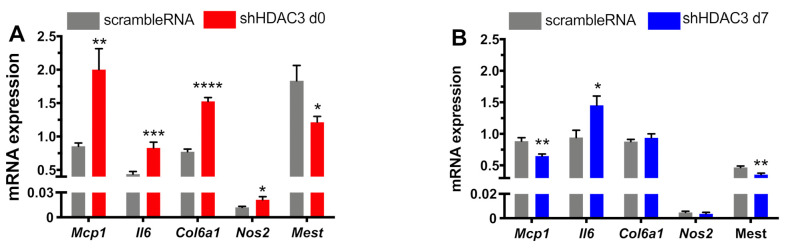
Expression of pro-inflammatory genes in C3H/10T1/2 cells infected with scramble/shHDAC3 at day 0 (**A**) or at day 7 (**B**) of differentiation to adipocytes. Cells silenced at day 0 were harvested at day 9 after induction of differentiation, while cells silenced at day 7 were harvested at day 11 after induction of differentiation. Data are presented as mean ± SEM. Statistical analyses were performed with GraphPad PRISM using unpaired Student’s *t*-test, * *p* < 0.05, ** *p* < 0.01, *** *p* < 0.001, **** *p* < 0.0001 (*n* = 9 from 3 independent experiments).

Inflammation is another important molecular aspect determining the adipocyte phenotype. Recent evidence indicates that expression of some inflammatory genes in adipocytes may be critical to maintain normal adipose tissue function [10,34]. Thus, we finally analyzed the expression profile of genes involved in adipose tissue inflammation in our experimental settings. In particular, the expression of monocyte chemoattractant protein-1 (*Mcp1*), a chemokine promoting monocyte/macrophage migration and infiltration, significantly increased when HDAC3 silencing occurred in adipocyte precursors (Figure 8A), while its expression significantly halved in HDAC3-silenced mature adipocytes (Figure 8B). The expression of pro-inflammatory cytokine interleukin 6 (Il6) was higher in adipocytes silenced at day 0 of differentiation (Figure 8A) and, similarly, although to a lower extent, in adipocytes silenced at day 7 (Figure 8B). We also analyzed the expression of collagen type 6a1 (*Col6a1*), which is enriched in adipocyte extracellular matrix and contributes to obesity-related inflammation of adipose tissue [35]. Its expression augmented with HDAC3 early silencing (Figure 8A), while no differences were observed when silencing occurred in differentiated adipocytes (Figure 8B). A similar expression profile was measured for inducible nitric oxide synthase (*Nos2*), important for cell signaling and involved in immune response initiation (Figure 8). Finally, we analyzed the expression of mesoderm-specific transcript (*Mest*), known as a regulator of adipocyte differentiation and size, but also involved in the adipocyte pro-inflammatory profile [36]. *Mest* expression significantly decreased with HDAC3 silencing at the beginning of differentiation (Figure 8A), while the decrease was milder with silencing at day 7 (Figure 8B).

### 2.5. HDAC3 Silencing in Precursor Cells Affects Gene Expression from Initial Stages of Adipocyte Differentiation

**Figure 9:**
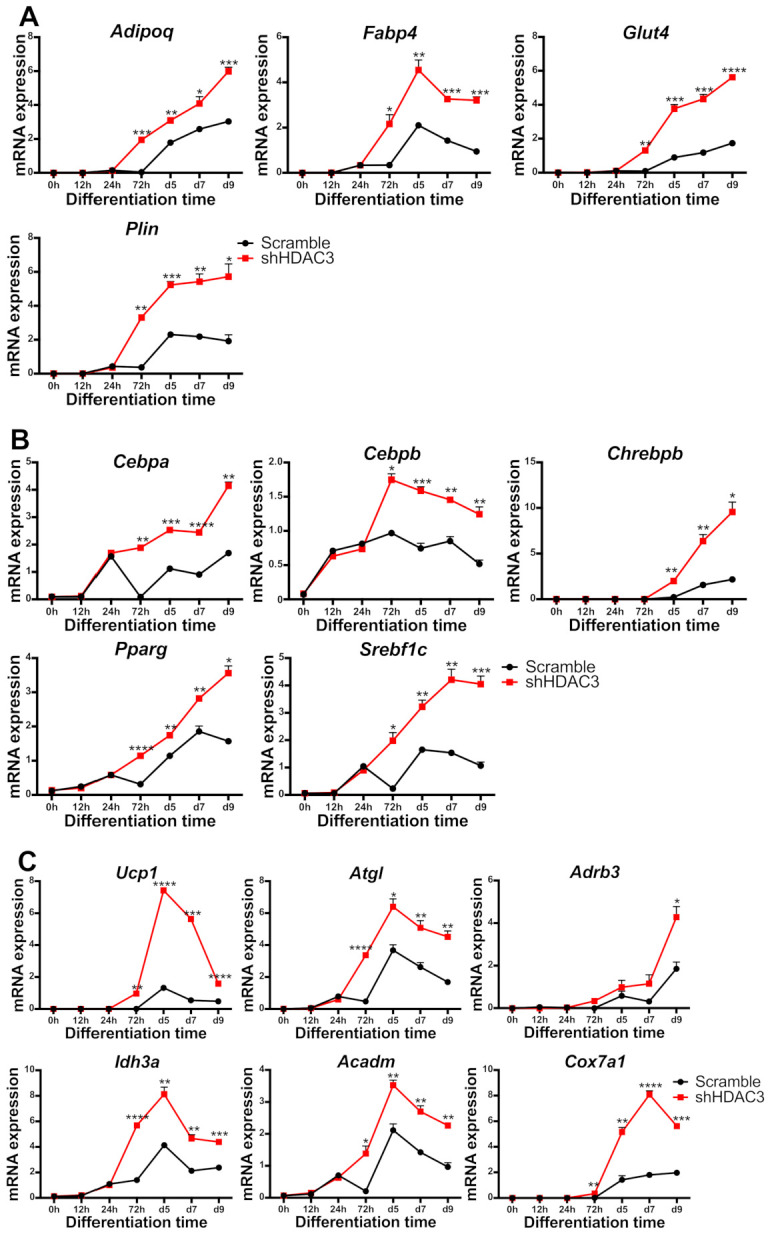
Kinetics of gene expression in C3H/10T1/2 cells infected with scramble/shHDAC3 and induced to differentiate to adipocytes. Expression of genes involved in adipocyte functionality (**A**), differentiation (**B**) and browning and mitochondrial oxidative metabolism (**C**). Data are presented as mean ± SEM of triplicate wells. Statistical analyses were performed using unpaired Student’s *t*-test, * *p* < 0.05, ** *p* < 0.01, *** *p* < 0.001, **** *p* < 0.0001.

To better characterize early events responsible for the effects of HDAC3 silencing, we performed a kinetic analysis of gene expression upon infection with shHDAC3 adenovirus. As shown in Figure 9, the expression of most of the abovementioned genes was significantly higher in HDAC3-silenced vs. scrambled shRNA adipocytes, as early as 72 h after induction of differentiation and silencing. *Adipoq*, *Plin*, *Glut4* and *Fabp4* gene expression was amplified 72 h after HDAC3 silencing (Figure 9A). Notably, the expression of transcriptional regulators involved in adipogenesis, *Cebpb*, *Srebf1c*, *Pparg* and *Cebpa* was also higher in differentiating adipocytes 72 h after HDCA3 silencing, while the enhanced expression of carbohydrate-responsive element-binding protein β (*Chrebpb*) was observed from day 5 (Figure 9B). This trend was defined for markers of browning, mitochondria functionality and metabolism such as *Ucp1*, *Idh3a*, *Cox7a1*, *Atgl* and *Acadm*. Furthermore, *Adrb3* showed higher expression from 72 h and its increase became significant at day 9 (Figure 9C).

Collectively, our results indicate that HDAC3 plays a crucial role during early stages of adipocyte differentiation and seems to act as a rheostat of the adipocyte phenotype (i.e., white vs. beige adipocyte phenotype).

## 3. Discussion

We previously showed that pharmacological inhibition of class I histone deacetylases using MS-275 improves obese phenotype and insulin sensitivity in both *db/db* [28] and diet-induced obese mice [29]. Furthermore, MS-275 amplifies the expression of genes important for adipocyte metabolism and browning in C3H/10T1/2 MSCs induced to differentiate to adipocytes [30]. Moreover, the metabolic effects of MS-275 were replicated in HDAC3-knockout mice in adipose tissue and in HDAC3-knockdown adipocytes [31].

Therefore, the aim of the present study was to understand whether the observed phenotypic effects were related to the actions of a specific class I HDAC in adipocyte precursors or in mature adipocytes. To this end, HDAC3 was silenced in MSCs induced to differentiate to adipocytes (day 0) and in already differentiated adipocytes (day 7). The effects of HDAC3 early silencing are consistent with the results obtained using the class I HDAC inhibitor MS-275. HDAC3 silencing at the beginning of differentiation promoted the expression of genes important for adipocyte functionality, browning and oxidative metabolism. Of note, in this study we showed that silencing of HDAC3 in C3H/10T1/2 adipocytes rewired metabolism and led to the concomitant increase of the lipogenic and lipolytic pathways in a futile cycle of fatty acid metabolism required to support the metabolic demand of adipocyte browning. These data consistently reaffirm our previous results obtained with [U-^13^C_16_]-palmitate, showing stimulation of de novo fatty acid synthesis and β-oxidation in C3H/10T1/2 adipocytes following HDAC3 silencing [31].

We also detected increased AKT phosphorylation, which suggests improved insulin signaling consequent to HDAC3 depletion in adipocytes. Interestingly, these results were accompanied by higher expression of the *Chrebpb* gene in adipose tissue, encoding a transcription factor that was previously shown to correlate with insulin sensitivity and possibly whole-body insulin action [33]. This transcription factor displays beneficial metabolic effects through the stimulation of glucose fate to de novo lipogenesis in adipocytes, thus integrating adipocyte and whole-body metabolism. Moreover, despite *Ppargc1a* expression decreasing in our model, the *Ppargc1b* isoform, whose expression was higher in HDAC3-silenced adipocytes, is known to exert compensatory actions in controlling mitochondrial energy metabolism [37]. In this regard, it should be noted that Enguix et al. [38] reported *Ppargc1b*-mediated effects seem to have limited impact on mitochondrial biogenesis while it appears to be important to maintain the expression of genes for mitochondrial oxidative pathways. Altogether, our results suggest the adipocyte phenotype is related to increased functionality rather than a greater number of mitochondria. Accordingly, mtDNA content did not change in silenced adipocytes, while functional assays confirmed higher oxidative capacity and uncoupling of the electron transport chain (ETC) from ATP synthesis.

**Figure 10:**
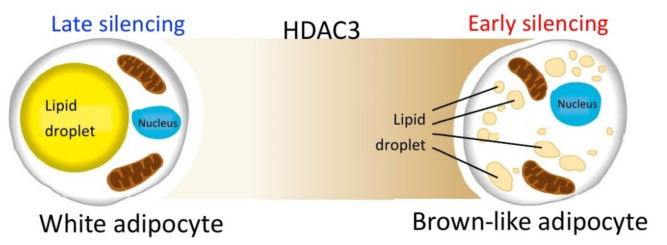
HDAC3 silencing during the early stages of differentiation is crucial for cell commitment toward the brown-like phenotype, characterized by enhanced oxidative metabolism.

On the other hand, no significant differences are measured when silencing occurs later during the differentiation process. These data suggest that the reduction of HDAC3 at early stages of differentiation, and possibly the related epigenome modifications, are crucial for cell commitment toward the brown-like phenotype, characterized by enhanced oxidative metabolism (Figure 10). Thus, the effects of class I HDAC inhibition or silencing might be exerted on adipocyte precursors rather than mature adipocytes. These conclusions are further corroborated by our kinetic analysis, which showed that expression of all the genes analyzed was increased starting from 24 h after induction of differentiation and silencing, suggesting HDAC3 impacts the adipocyte phenotype from the very first hours of differentiation. Based on our previous results, we predict that the molecular basis of HDAC3 early silencing might be similar to those demonstrated in CH3/10T1/2 adipocytes treated with MS-275 [30]. MS-275 was shown to increase H3K27 acetylation in *Pparg* and *Ucp1* enhancer regions as early as 12 h after the beginning of differentiation and consequently to upregulate the expression of these key genes by acting on early chromatin remodeling of their enhancer regions [30]. It is tempting to speculate that the early increased expression of transcription factors involved in adipogenesis, such as *Pparg*, *Cebpa, Cebpb*, *Chrebpb* and *Srebf1*, might be target of possible epigenetic effects related to HDAC3 silencing at the beginning of differentiation. More experiments will be necessary in the future to probe this hypothesis.

Finally, the characterization of the role of HDAC3 on the adipocyte phenotype was extended to markers of inflammation, as we analyzed the expression of pro-inflammatory genes when HDAC3 was silenced at the beginning or at the end of differentiation. The expression of *Mcp1*, *Il6*, *Col6a1* and *Nos2* significantly increased in HDAC3-early-silenced adipocytes, and this observation is consistent with previous reports showing that low-grade inflammation in adipocytes is important for physiological adipose tissue expansion and remodeling and metabolic health [10,34]. In fact, expression of the analyzed genes increased only around 2-fold as compared to controls, while these markers usually greatly increase upon acute pro-inflammatory stimuli. In this regard, it is crucial to distinguish between inflammation originating in adipocytes and inflammation stemming from immune cells within adipose tissue. The former seems a key prerequisite for the proper function of adipocytes, assuring healthy adipose tissue expansion and remodeling. In addition, transient acute inflammation plays a beneficial role in healthy adipogenesis and metabolic homeostasis, while suppressed adipocyte inflammation has been shown to lead to systemic metabolic disturbances [10]. Therefore, adipose tissue inflammation could be an adaptive response that enables safe storage of excess nutrients. Furthermore, the suppression of adipocyte inflammation impairs adipose tissue function and promotes insulin resistance [34]. According to this view, inflammation in adipocytes could be considered as a protective adaptive response to potentially dangerous cues and it is critical to resolve tissue damage by initiating a repair process to reestablish tissue structure and function. Within this frame, we believe the increased expression of some inflammatory genes observed in HDAC3-silenced adipocytes, along with the metabolic rewiring and oxidative phenotype, could be viewed as a beneficial improvement of the adipocyte phenotype. The moderate upregulation of inflammatory marker genes may prelude to a later increase of the corresponding factors that may be detected only at later stages of inflammation onset. Thus, the increased expression of inflammatory genes could be regarded as an early marker of low-grade inflammation arising from adipocytes. Moreover, the expression of *Mest*, a factor involved in unhealthy adipose tissue expansion in an obesogenic environment [39], significantly decreased upon HDAC3 silencing, consistent with its role in the regulation of adipocyte differentiation and inflammation. On the other hand, the expression of pro-inflammatory genes did not increase with HDAC3 silencing at day 7, resulting in lower adipocyte differentiation and functionality as opposed to early silencing of HDAC3.

In conclusion, our results demonstrate HDAC3 action during the very early stages of cell differentiation is essential to determine their phenotypic fate. Although the mechanistic basis of the effects of HDAC3 silencing needs to be investigated to delineate the key molecular events involved in the process, our results provide new evidence of the role played by a specific HDAC in determining the metabolic features of fully differentiated adipocytes.

## 4. Materials and Methods

### 4.1. Cell Culture and Differentiation

C3H/10T1/2, Clone 8 (ATCC^®^ CCL-226™, Manassas, VA, USA) were cultured in Dulbecco’s Modified Eagle Medium (DMEM, Sigma-Aldrich, St. Louis, MO, USA) supplemented with 10% heat-inactivated fetal bovine serum (FBS, Euroclone, Pero, Italy), 1% L-glutamine (Life Technologies Italia, Monza, Italy) and 1% penicillin-streptomycin (Life Technologies Italia, Monza, Italy). Differentiation was induced using growth medium with 5 μg/mL insulin (Sigma-Aldrich, St. Louis, MO, USA), 0.5 mM 3-isobutyl-1-methylxanthine (Sigma-Aldrich, St. Louis, MO, USA), 2 μg/mL dexamethasone (Sigma-Aldrich, St. Louis, MO, USA) and 5 μM rosiglitazone (Cayman Chemicals, Ann Arbor, MI, USA). For differentiation, cells were cultured in induction medium for 3 days and maintained in growth medium with 5 μg/mL insulin for 6 to 8 days (Figure 1) or as indicated in each figure legend. At the end of differentiation images of cells were captured with an Axiovert microscope (Carl Zeiss, Milano, Italia) at 10× magnification. Adipocytes were manually counted according to morphology and lipid accumulation.

### 4.2. Cell Infection

To silence *Hdac3*, 100 MOI shHDAC3 adenovector (Vector BioLabs, Malvern, PA, USA) was added to induction medium at day 0 or at day 7 to maintenance medium. Cells infected with 100 MOI Scramble were used as negative control (Figure 1). Infected cells were harvested at the end of differentiation. For kinetic experiments cells were harvested during differentiation, after 12 h, 24 h, 72 h, 5 days, 7 days and 9 days from induction.

### 4.3. Gene Expression

Total RNA from cells cultured in 12-well plates was isolated using NucleoSpin RNA kit (Macherey-Nagel, Dueren, Germany) and quantified with Nanodrop (Life Technologies Italia, Monza, Italy). Real-time quantitative PCR was performed using iScriptTM One Step RT PCR for Probes (Bio-Rad Laboratories, Segrate, Italy), following the manufacturer’s instructions. Primers and probes were obtained from Eurofins Genomics Italy (Vimodrone, Italy). The complete list is reported in Table 1. Specific mRNA amplification was normalized to 36B4 mRNA and quantified with the standard curve method. Data from three independent experiments were included for gene expression analyses.

### 4.4. Lipid Content in Adipocytes

Lipid content in C3H/10T1/2 adipocytes was quantified using Oil Red O (ORO, Sigma-Aldrich, St. Louis, MO, USA) staining. ORO was extracted from stained samples in wells with 1.5 mL isopropanol via incubation for 10 min, and absorbance was measured at 500 nm.

### 4.5. mtDNA Quantification

DNA from cells cultured in 12-well plates was isolated using Nucleospin Tissue (Macherey-Nagel, Dueren, Germany) and quantified with Nanodrop (Life Technologies Italia, Monza, Italy). Real-time quantitative PCR was performed using an iScriptTM One-Step RT-PCR Kit for Probes (Bio-Rad Laboratories, Segrate, Italy) following the manufacturer’s instructions and omitting iScript. Primers and probes of mtCox2 were obtained from Eurofins Genomics Italy (Vimodrone, Italy) (Table 1). A DNA quantity of mtCox2 was normalized to 36B4 content. Data from three independent experiments were included for the analysis.

### 4.6. Glycerol Release from Adipocytes

Cell culture medium from C3H/10T1/2 adipocytes grown and differentiated in 12-well plates was collected at the end of the experiment. Glycerol release in cell culture medium was measured with a Glycerol Assay Kit (Sigma-Aldrich, St. Louis, MO, USA) as an index of fatty acid β-oxidation.

### 4.7. Measurement of Mitochondrial Respiration

Mitochondrial respiration was assessed in mature adipocytes using Seahorse XFe24. A Cell Mito Stress Test (Agilent Technologies, Santa Clara, CA, USA) was performed following the manufacturer’s instruction and serially injected with 1.5 µM oligomycin, 1 µM carbonyl cyanide-4-(trifluoromethoxy)phenylhydrazone (FCCP) and 0.5 µM rotenone/antimycin A. Cells were seeded in Seahorse culture plates coated with gelatin (0.1% *w*/*v*). After infection with scramble or shHDAC3 (Day 0), cells were differentiated until day 9. On the day of assay, the Seahorse assay medium was supplemented with 10 mM glucose and 2 mM glutamine (pH 7.4). After assay performance, cells were lysed in RIPA buffer and proteins were quantified using BCA assay (Euroclone, Pero, Italy). Seahorse data were normalized to the total amount of proteins (μg). Data were analyzed using Wave software, including the Seahorse XF Mito Stress Test Report Generator (Agilent Technologies, Santa Clara, CA, USA).

### 4.8. Protein Analyses and Quantification

Cells cultured in 6-well plates were lysed using Laemmli buffer. Lysates were homogenized with TissueLyser (Qiagen, Milano, Italy) and heated to 99 °C. Proteins were separated by SDS-PAGE and transferred to nitrocellulose membranes (GE Healthcare Life Sciences, Chicago, IL, USA). Sample loading and transfer was checked via Ponceau staining (Sigma-Aldrich, St. Louis, MO, USA). Membranes were blocked using 5% bovine serum albumin (Sigma-Aldrich, St. Louis, MO, USA) in TBS with 0.1% Tween 20 (Sigma-Aldrich, St. Louis, MO, USA). Membranes were incubated with antibodies against HDAC3 (Santa Cruz Biotechnology, Dallas, TX, USA), phospho-AKT Ser473 and pan-AKT (Cell Signaling Technology, Danvers, MA, USA), antibody cocktail to detect mitochondrial proteins (Total OXPHOS Rodent WB Antibody Cocktail, Abcam, Cambridge, UK), PPARγ (Cell Signaling Technology, Danvers, MA, USA) or HSP90 antibody (Santa Cruz Biotechnology, Dallas, TX, USA) as a loading control. HRP-conjugated secondary antibodies were used for detection with chemiluminescence (Pierce ECL Western Blotting Substrate, Life Technologies Italia, Monza, Italy). Protein levels were quantified and normalized to HSP90 using ImageJ software (National Institutes of Health, Bethesda, MD, USA).

### 4.9. Statistical Analyses

Statistical analyses were performed using the unpaired two-tailed Student *t*-test with Prism (GraphPad, San Diego, CA, USA).

## Figures and Tables

**Table 1:** Sequences of primers and probes used for gene expression and mtDNA quantification analyses. *Prdm16* and *Adrb3* genes were analyzed using ABI mix (Life Technologies Italia, Monza, Italy).

Gene		5′→3′ Sequence
*Hdac3*	Forward	TGTCTCAATGTGCCCTTACG
	Reverse	CCTAATCGATCACAGCCCAG
	Probe	ACTTCTACCAGCCGACGTGCATC
*Pparg*	Forward	TGTTATGGGTGAAACTCTGGG
	Reverse	AGAGCTGATTCCGAAGTTGG
	Probe	CCCTCGCTGATGCACTGCCTATGA
*Cebpa*	Forward	AGAGCCGAGATAAAGCCAAAC
	Reverse	TCATTGTCACTGGTCAACTCC
	Probe	AGCACCTTCTGTTGCGTCTCCA
*Cebpb*	Forward	CCCCGCGTTCATGCA
	Reverse	CAGTCGGGCTCGTAGT
	Probe	ACTTCCATGGGTCTAAAGGCG
*Chrebpb*	Forward	CTGCAGATCGCGTGGAG
	Reverse	GCAACTTGAGGCCTTTGAAG
	Probe	CAAGCTGGTCTCTCCCAAGTGGAA
*Srebf1c*	Forward	ATGGATTGCACATTTGAAGACATGCT
	Reverse	CCTGTGTCCCCTGTCTCAC
	Probe	CTTCCCGGGCCTGTTTGACGCCCCCTA
*Adipoq*	Forward	AGGCATCCCAGGACATC
	Reverse	CCTGTCATTCCAACATCTCC
	Probe	CCTTAGGACCAAGAAGACCTGCATCTC
*Plin*	Forward	ACAGACACAGAGGGAGAGG
	Reverse	AGTGTTCTGCACGGTGTG
	Probe	AGGAGGAAGAAGAGTCCGAGGCT
*Fabp4*	Forward	GGCGTGGAATTCGATGAA
	Reverse	GCTTGTCACCATCTCGTT
	Probe	TGATGCTCTTCACCTTCCTGTCGT
*Glut4*	Forward	TGTCGCTGGTTTCTCCAACTG
	Reverse	CCATACGATCCGCAACATACTG
	Probe	ACCTGTAACTTCATTGTCGGCATGGGTTT
*Ucp1*	Forward	GAGCTGGTAACATATGACCTC
	Reverse	GAGCTGACAGTAAATGGCA
	Probe	ACAAAATACTGGCAGATGACGTCCC
*Ppara*	Forward	ACGCATGTGAAGGCTGTAAG
	Reverse	CACTTGTGAAAACGGCAGTAC
	Probe	CGGCTGAAGCTGGTGTACGACAA
*Cidea*	Forward	CACGCATTTCATGATCTTGG
	Reverse	CCTGTATAGGTCGAAGGTGA
	Probe	TTACTACCCGGTGTCCATTTCTGTCC
*Lpl*	Forward	GCCATGACAAGTCTCTGAAG
	Reverse	CTTTCAAACACCCAAACAAGG
	Probe	AGTCTGGCTGACACTGGACAAACA
*Atgl*	Forward	TCGTGTTTCAGACGGAGA
	Reverse	CACATAGCGCACCCCT
	Probe	TGCAGACATTGGCCTGGATGAG
*Cd36*	Forward	GCGACATGATTAATGGCACAG
	Reverse	GATCCGAACACAGCGTAGATAG
	Probe	CAACAAAAGGTGGAAAGGAGGCTGC
*Cpt1b*	Forward	GATGCAGTTCCAGAGAATCC
	Reverse	CTTGTTCTTGCCAGAGCT
	Probe	TCTGCCCACTCTACCCTTCCTC
*Slc25a20*	Forward	TCTTTGGGTTTGGTCTGGG
	Reverse	ATTTGATCCGTTCTCCAGGG
	Probe	TCTCCAGAGGATGAACTTAGCTACCCAC
*Hadh*	Forward	TCTTGACTATGTTGGACTGGATAC
	Reverse	AAGGACTGGGCTGAAATAAGG
	Probe	CTTGGACGGGTGGCATGAAATGG
*Acadm*	Forward	ACCCAGATCCTAAAGTACCC
	Reverse	CGAAAGCAATTCCTCTGGTG
	Probe	TGGCCCATGTTTAGTTCCTTTTTTCCAA
*Acox1*	Forward	TCACGTTTACCCCGGC
	Reverse	CAAGTACGACACCATACCAC
	Probe	CATCAAGAACCTGGCCGTCTGC
*Ppargc1b*	Forward	GGTGTTCGGTGAGATTGTAGAG
	Reverse	GTGATAAAACCGTGCTTCTGG
	Probe	TCTTTTACTTCTCGTCAGCACCTGGC
*Ppargc1a*	Forward	CATTTGATGCACTGACAGATGGA
	Reverse	GTCAGGCATGGAGGAAGGAC
	Probe	CCGTGACCACTGACA ACGAGGCC
*Idh3a*	Forward	ACGGAAGGAGAATACAGTGG
	Reverse	GTACTCGAAGGCAAACTCTG
	Probe	ACCCCATCAACGATCACATGCTCA
*mtCox2*	Forward	TGGTGAACTACGACTGCT
	Reverse	CTGGGATGGCATCAGTTT
	Probe	TGGCAGAACGACTCGGTTATCAACT
*Cox6a1*	Forward	GTTCGTTGCCTACCCTCAC
	Reverse	TCTCTTTACTCATCTTCATAGCCG
	Probe	ACCATACCCTCTTCCACAACCCTCA
*Cox7a1*	Forward	TGTGGCAGAGAAGCAGAAG
	Reverse	AGCCCAAGCAGTATAAGCAG
	Probe	CGACAATGACCTCCCAGTACACTTGA
*Suclg1*	Forward	AATGATCCAGCCACAGAAGG
	Reverse	AGCAATGAAGGACACTACAGG
	Probe	AGCATAACTCAGGTCCAAAGGCCAA
*Mcp1*	Forward	CTCATGTACCCGCTGTATAG
	Reverse	TTAGAGCCACGACCATACA
	Probe	ATACTGGATGCCGTCTATGTCG
*Il6*	Forward	ACGATAGTCAATTCCAGAAACC
	Reverse	GTTGTCACCAGCATCAGT
	Probe	CTTGCAGAGAGGAACTTCATAGC
*Col6a1*	Forward	CTGGTGAAGGAGAACTATGCAG
	Reverse	GTCTAGCAGGATGGTGATGTC
	Probe	CCGGGGAGGAGAATGTGATTGGA
*Mest*	Forward	TTCTGTGTCCATCCCCATTC
	Reverse	CTGTGGGTAGTGGCTAATGTG
	Probe	TGTACAGGAAAACGCTGCCGC
*Nos2*	Forward	CCCTCCTGATCTTGTGTTGGA
	Reverse	CAACCCGAGCTCCTGGAA
	Probe	CCATGGAGCATCCCAAGTACGAGT

## Data Availability

The data presented in this study are available upon request from the corresponding author.

## Abbreviations

HDACs	Histone deacetylases
HDAC3	Histone deacetylase 3
MSCs	Mesenchymal stem cells
BMI	Body mass index
CREB	cAMP response element binding protein
C/EBP	CCAAT/enhancer-binding protein
SREBP-1c	Sterol regulatory element binding protein-1c
PPARG	Peroxisome proliferator-activated receptor gamma
WAT	White adipose tissue
BAT	Brown adipose tissue
UCP1	Uncoupling protein 1
ETC	Electron transport chain
FCCP	carbonyl cyanide-4-(trifluoromethoxy)phenylhydrazone
ChREBPB	Carbohydrate-responsive element-binding protein
PLIN	Perilipin
FABP4	Fatty acid binding protein 4
ADIPOQ	Adiponectin
GLUT4	Glucose transporter 4
CD36	Cluster of differentiation 36
LPL	Lipoprotein lipase
ATGL	Adipose triglyceride lipase
CPT1B	Carnitine palmitoyl-transferase 1b
SLC25A20	Solute carrier family 25 member 20
HDAH	Hydroxyacyl-coenzyme A dehydrogenase
ACADM	Medium-chain acyl-CoA dehydrogenase
ACOX1	Peroxisomal acyl-coenzyme A oxidase 1
ADRB3	β3 adrenergic receptor
PPARA	Peroxisome proliferator activator receptor α
CIDEA	Cell death inducing DFFA-like effector α
PRDM16	PR domain containing 16
IDH3A	Isocitrate dehydrogenase subunit α
mtCOX2	Mitochondrial cytochrome c oxidase subunit 2
COX6A1	Cytochrome c oxidase subunit 6a1
COX7A1	Cytochrome c oxidase subunit 7a1
SUCLG1	Succinyl-CoA ligase subunit 1
PPARGC1A	PPARγ coactivator 1α
PPARGC1B	PPARγ coactivator 1β
MCP1	Monocyte chemoattractant protein-1
IL6	Interleukin 6
COL6A1	Collagen type 6a1
iNOS	Inducible nitric oxide synthase
MEST	Mesoderm-specific transcript
H3K27	Lysine 27 Histone 3
